# First detection of *Leishmania killicki *(Kinetoplastida, Trypanosomatidae) in *Ctenodactylus gundi *(Rodentia, Ctenodactylidae), a possible reservoir of human cutaneous leishmaniasis in Tunisia

**DOI:** 10.1186/1756-3305-4-159

**Published:** 2011-08-11

**Authors:** Kaouther Jaouadi, Najoua Haouas, Dhekra Chaara, Mohamed Gorcii, Najla Chargui, Denis Augot, Francine Pratlong, Jean-Pierre Dedet, Selim Ettlijani, Habib Mezhoud, Hamouda Babba

**Affiliations:** 1 Laboratoire de Parasitologie-Mycologie (99UR/08-05), Département de biologie clinique, Faculté de Pharmacie de Monastir, Tunisia; 2 JE 2533 - USC ANSES «Transmission vectorielle et épidémiosurveillance de maladies parasitaires (VECPAR)», Université de Reims Champagne-Ardenne, 51 rue Cognacq-Jay, 51096 Reims, France; 3 Université Montpellier 1, Centre National de référence des Leishmania, UMR MIVEGEC (UM1, CNRS 5290, IRD 224), Laboratoire de Parasitologie-Mycologie, CHRU de Montpellier, France; 4 Direction des Soins de santé de Base, Gafsa, Tunisia

## Abstract

**Background:**

*Leishmania killicki *was originally described in 1980 in southeast Tunisia. It was also recently reported in Lybia and Algeria. Nevertheless, neither vector nor reservoirs of this parasite are known. The identification of the vector and the animal reservoir host of *L. killicki *is critical for the establishment of an efficient control strategy.

**Findings:**

blood, popliteal lymph node, spleen, bone marrow, liver and skin were collected from 50 rodents in 2009 in south western Tunisia. Samples were smeared onto glass slides, cultured on NNN medium and tested by polymerase chain reaction for *Leishmania *detection. Parasites were detected by PCR from 10 *Psammomys obesus *and from two *Ctenodactylus gundi*. Parasite identification was performed simultaneously by internal transcribed spacer 1 PCR-RFLP and by PCR sequencing. Both *Leishmania major *and *Leishmania killicki *were identified from infected *Psammomys *and *Ctenodactylus gundi *respectively.

**Conclusion:**

This is the first report of *Leishmania killicki *identified from *Ctenodactylus gundi *in Tunisia. This result supports the assumption that *C. gundi *is a potential reservoir for *Leishmania killicki*.

## Findings

In Tunisia, *Leishmania infantum*, *L. major *and *L. killicki *are responsible for cutaneous leishmaniasis (CL). This last taxon has been described in 1980 in Southeast Tunisia, based on the identification of 29 human strains [1]. It is responsible for CL with a chronic evolution of the lesion and a low endemicity (about 10 cases per year). Since 2004, new foci of CL caused by *L. killcki *have emerged in different parts of the country. *Leishmania killicki *was also recently reported in Lybia and in Algeria, two neighbouring countries of Tunisia [2-4]. Nevertheless, until recently, little information was available on the epidemiology of *L. killicki *CL and neither the vector nor reservoir hosts were known.

The collection of wild animals for detection of a possible parasitic infection seems to be the most effective method for reservoir host identification. Therefore, an epidemiological study was carried out in Metlaoui province in Southwestern Tunisia, a new emerging focus of this taxon, with the objective of detecting and characterizing *Leishmania killicki *infection in wild rodents.

**Figure 1:**
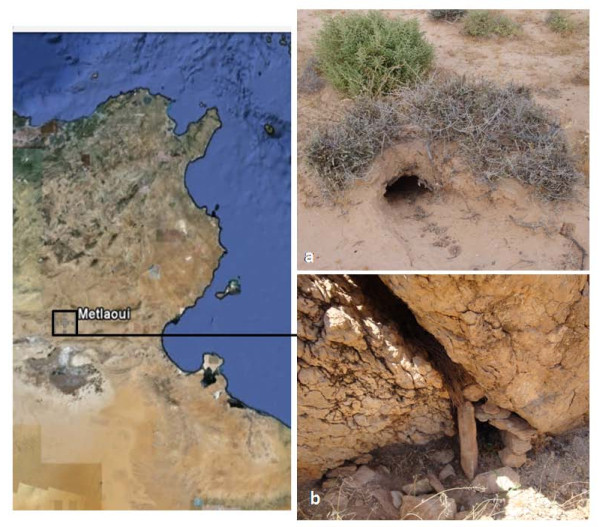
**Geographical situation and landscape of rodent collection sites**. The map of Tunisia on the left shows the geographical situation of Metlaoui province, the study area. The photos on the right show rodent burrow, the biotope of *Psammomys obesus *(a) and crevices within boulder, the biotope of *Ctenodactylus gundi *(b) where rodent traps were set.

Wild rodents including *Psammomys *(*Ps*.) *obesus, Ctenodactylus *(*C*.) *gundi, Meriones *(*Mr*.) *shaw*i and *Mus *(*M*.) *musculus *were trapped alive, in 2009 in Metlaoui Province South western Tunisia, by using home-made metal rodent traps covered up with sand and placed at burrow entrances and rock crevices (Figure 1). Trapped rodents were examined for the existence of skin lesions. After euthanizing, samples of blood, liver, spleen, bone marrow, lymph nodes and skin lesion (if present) were obtained for parasite detection. In some animals, samples were not available from all sites. The study that concerned these rodents was conducted adhering to the American Psychological Association's Guidelines for Ethical Conduct in the Care and Use of Animals. The first part was smeared onto a glass slide, fixed with methanol, stained with Giemsa, and examined by microscopy. The second part was inoculated on Novy-MacNeal-Nicolle medium. The cultures were incubated at 26 °C and observed every week for 1 month. The third part was used for DNA extraction. DNA from all tissues was tested for *Leishmania spp*. infection by internal transcribed spacer 1 (ITS1) PCR according to the protocol of Schönian et al., 2003 [5]. Positive PCR products were analysed by both: i) the Restriction Fragment Length polymorphism (RFLP) method [5], and ii) by sequencing using the same primers than those used for the PCR (Eurofins MWG Operon, Munich, Germany).

**Figure 2:**
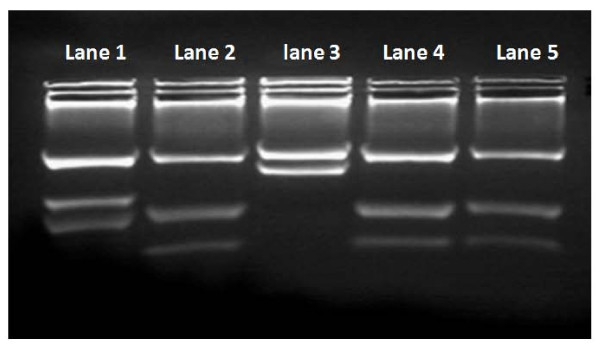
**Restriction pattern of the amplified ribosomal Internal Transcribed Spacer 1 using HaeIII**. Reference strains used were: *Leishmania infantum *MHOM/FR/78/LEM75 (Lane 1) (three fragments of 187 bp, 72 bp and 55 bp), *Leishmania killicki *MHOM/TN/LEM163 (Lane 2) (three fragments of 188 bp, 57 bp and 26 bp) and *L. major *MHOM/MA/81/LEM265 (Lane 3) (two fragments of 206 bp and 132 bp). Restriction pattern of the PCR products amplified from the two *Ctenodactylus gundi *bone marrow (Lane 4 and 5) are the same as *Leishmania killicki *profile (Lane 2).

In total, 50 rodents were caught, belonging to the following species: *Ps. obesus *(n = 40), *C. gundi *(n = 6), *Mr. shawi *(n = 3) and *M. musculus *(n = 1). Among the 205 tissue samples collected from these rodents, only the bone marrow of a single *C. gundii *was positive by direct examination, but culture was negative for all organs. Sixteen organs were detected positive for *Leishmania spp*. infection using ITS1-PCR: they were from ten *Ps. obesus *(cutaneous lesion n = 5, bone marrow n = 3, lymph node n = 3, spleen n = 3) and from two *C. gundi *(bone marrow). The analysis of the positive PCR products by RFLP showed that all positive organs sampled from *Ps. obesus *were infected with *L. major*. The restriction patterns of the two positive PCR products from the *C. gundii *bone marrow were identical to the *L. killicki *reference strain profile (Figure 2). Sequencing of the positive PCR products has shown the same result as RFLP analysis.

If the detection of *L. major *in *Ps. obesus *was an expected result, since this rodent was already described as the reservoir of this *Leishmania *taxon [6], our study is the first report of *L. killicki *infecting a rodent. The detection of this *Leishmania *species in the bone marrow of asymptomatic animals indicates that it can visceralize in *C. gundi*. Consequently, this rodent species could be a natural host of *L. killicki*, and an efficient reservoir host as two out of six specimens were found infected.

*Ctenodactylus gundi *is a gregarious rock-dwelling rodent found in northern Africa. In Tunisia it exists in the mountainous area of Tataouine (the original focus of *L. killicki*) [7] as well as in all emerging Tunisian foci of CL caused by *L. killicki *[8]. *C. gundi *populations inhabit crevices within boulder mounds in the vicinity of villages. The rock crevices and caves present suitable breeding sites for sand flies. This rodent species was suspected to be the *L. killicki *reservoir since the end of the last century [7,9,10]; however no evidence has been given before. The detection of *L. killicki *in *C. gundi *by both direct examination and PCR method is the first proof of the potential role of this wild rodent as a natural host of this parasite.

## Conclusion

Cutaneous leishmaniasis due to *L. killicki *seems to be a zoonotic disease involving *C. gundi *in its life cycle. However, the isolation of the parasite from this rodent is crucial for the confirmation of this first result. Further investigation, such collecting wild rodents in other *L. killicki *foci, is required to discern the potential epidemiologic role of *Ctenodactylus gundi *in spreading infection.

## Competing interests

The authors declare that they have no competing interests

## Authors' contributions

Conceived and designed the experiments: HB, NH, KJ, DA, FP and JPD. Performed the experiments: KJ, DC, NH, NC, HM. Drafted the manuscript: NH, KJ, HB, FP, JPD. Participated in field missions: KJ, DC, MG, DA, HB, NH. All authors approved the final version of the manuscript.
